# Dengue Fever Accompanied by Neurological Manifestations: Challenges and Treatment

**DOI:** 10.7759/cureus.60961

**Published:** 2024-05-23

**Authors:** Jay P Patel, Faizanali Saiyed, Daksh Hardaswani

**Affiliations:** 1 Research, Chirayu Medical College and Hospital, Bhopal, IND; 2 Internal Medicine, Odessa National Medical University, Odessa, UKR; 3 Research, Jawaharlal Nehru Medical College, Wardha, IND

**Keywords:** dengue-related hospitalization, evolution of dengue, dengue with warning signs, dengue fever/complications, dengue complication, dengue viruses, dengue encephalitis, neurological manifestations of dengue, expanded dengue synrome, dengue fever (df)

## Abstract

Dengue, commonly referred to as 'breakbone fever,' is a mosquito-borne arboviral infection transmitted by *Aedes aegypti*, featuring an average incubation period of approximately seven days. Key cytokines such as interferon-gamma (IFN-γ), tumor necrosis factor (TNF)-α, and interleukin (IL)-10 are pivotal in the pathogenesis of dengue. Travelers are particularly susceptible to contracting dengue fever, with disease severity often associated with CD8+ T cell response. Without proper hospitalization during severe cases like dengue hemorrhagic fever (DHF)/dengue shock syndrome (DSS), mortality rates can escalate to 50%. Dengue fever can lead to various complications, including neurological manifestations such as encephalopathy, encephalitis, cerebral venous thrombosis, myelitis, posterior reversible encephalopathy syndrome, strokes (both ischemic and hemorrhagic), immune-mediated neurological syndromes (such as mononeuropathy, acute transverse myelitis, Guillain-Barre syndrome, and acute disseminated encephalomyelitis), and neuromuscular complications. Treatment protocols typically involve assessing disease activity using composite indices, pursuing treatment objectives, and administering intravenous fluids according to symptomatology. Given the absence of specific antiviral treatment for dengue, supportive care, particularly hydration, remains paramount during the early stages. It is crucial to recognize that dengue viruses may contribute to the development of neurological disorders, particularly in regions where dengue is endemic. Furthermore, there is a necessity for well-defined criteria for specific neurological complications. Primary prevention strategies primarily revolve around vector control measures, which play a critical role in curtailing the spread of dengue.

## Introduction and background

Dengue is a mosquito-transmitted arboviral infection, primarily by Aedes aegypti and to some extent by albopictus, with an average incubation period of seven days, showcasing symptoms such as headache, fever, myalgia, joint pain, and exanthem [1]. It is also known as "breakbone fever" and falls under the genus flavivirus [2]. This positive strand-RNA virus is enclosed within a protein capsid surrounded by an envelope, giving it a spherical shape. It comprises four serological types, extending to critical forms of dengue hemorrhagic fever/dengue shock syndrome (DHF/DSS) [2, 3].

Dengue has been recognized for over 200 years and is prevalent in Asia, along the Atlantic and Gulf coasts of the United States and the Caribbean, first acknowledged in the Philippines in 1954 [2]. Concurrent infections occur at a rate ranging from 2.5% to 30%, escalating to 40-50% in highly susceptible dengue areas [3]. Travelers are more vulnerable to dengue illnesses and are likely to contract the dengue virus from endemic countries [3, 4]. Upon return to their home countries, if individuals are in the viremic phase, they may transmit new serotypes to non-endemic nations [4]. Abnormal laboratory tests such as thrombocytopenia, neutropenia, and elevated liver function tests are common [5].

Dengue complication rates range from 0.9% to 3% [5]. It causes an estimated 390 million infections, 100 million clinically evident cases, and 500,000 presentations of severe dengue annually worldwide, with at least 2.5 billion individuals at risk. Currently, dengue is the most prevalent arthropod-borne virus globally [6]. A portion of severe disease has been associated with elevated levels of cytokines, including interferon-gamma (IFN-γ), tumor necrosis factor (TNF)-γ, and interleukin (IL)-10 [7]. Disease severity has also been linked to the activation of CD8+ T cells and the growth of serotype-reactive low-affinity dengue virus (DENV)-specific T cells, which generate high amounts of vasoactive cytokines. The capillary leak syndrome linked to DHF may be influenced by a pathologic cytokine response following significant T-cell activation [8,9]. Without hospitalization, the mortality rate during the DHF/DSS phase might reach 50% [9].

Neurological complications associated with DENV infection are increasingly recognized, some posing significant risks if not promptly addressed. These manifestations encompass various conditions, including dengue virus encephalopathy and encephalitis, immune-related disorders such as acute disseminated encephalomyelitis, myelitis, Guillain-Barre syndrome, brachial neuritis, and acute cerebellitis, among others. Additionally, neuromuscular issues like hypokalemic paralysis, transient benign muscle dysfunction, and myositis can occur. Furthermore, dengue-related stroke and cerebral venous thrombosis represent potential, albeit less common, outcomes [10, 11].

Dengue virus (DENV) is a neurotropic virus capable of infecting central nervous system (CNS)-supporting cells. During the acute stage of the infection, neural damage is caused by direct neuro-invasion or antibody-dependent enhancement [10]. Headache is the most common symptom, affecting more than 90% of patients, and can be localized or generalized [12]. DENV-2 and DENV-3 are the main culprits for neurological conditions. These serotypes have been detected in cases of encephalitis, meningitis, and myelitis in patients with dengue fever. DENV-4 was also found in brain cells and cerebrospinal fluid (CSF) of a patient with encephalitis, determined by immunohistochemistry [13, 14].

Currently, there is no vaccine or approved antiviral drug; primary treatment is with supportive fluids [15]. Direct antiviral medicines that lessen dengue severity might be beneficial; however, they would need to suppress all four virus serotypes successfully. In this article, we will see whether the antiviral drug is effective in suppressing dengue's seriousness [15]. Vector control is still the most extensively used method for preventing dengue spread. Three significant ways to tackle vector control are chemical, environmental, and biological [16]. Neurological manifestations nowadays are observed and appear as a challenge for medical practice. This study will review the neurological complications, focusing on a better understanding of the disease and its treatment.

## Review

Methodology

A narrative literature review was conducted utilizing resources from the World Health Organization (WHO) and the Centers for Disease Control and Prevention (CDC), alongside electronic databases such as PubMed and Google Scholar. The search was aimed at identifying relevant studies, reviews, and reports on dengue fever with a specific focus on neurological manifestations and their associated challenges and treatments. The initial search yielded a substantial number of articles. Articles included in this review are from 1989-2023. Titles and abstracts of these articles were screened for relevant topics.

Dengue fever typically lasts five to seven days and is characterized by self-limiting fever, which can be debilitating during the acute stage. Clinical symptoms vary according to the patient's age. Infants and young children may present with undifferentiated febrile illness and maculopapular rash, while older children and adults may experience moderate or severe illness, featuring high fever (biphasic), severe headache, retroorbital discomfort, myalgia, arthralgia, nausea, vomiting, and petechiae. Leukopenia and thrombocytopenia are common across all age groups. In some cases, dengue fever may be accompanied by bleeding complications such as gingival bleeding, epistaxis, gastrointestinal bleeding, haematuria, and menorrhagia (in women) [17].

Dendritic cells (DCs) play a crucial role as mediators between innate and adaptive immune responses during viral invasion [18]. They upregulate pro-inflammatory cytokines and co-stimulatory molecules, thereby stimulating human immune responses [19]. However, NS1 can limit the maturation and migration of DCs, thereby hindering the induction of IFN-γ release from Th1 cells by regulating gene expression [19]. Effective inactivation of the influenza virus NS1 requires antiviral drugs capable of restoring host antiviral responses, including innate immunity associated with IFN production and restricting virus replication [20]. NS1 of dengue virus (DENV) can facilitate immune evasion by engaging with relevant complement components in different complement activation pathways, such as classical and lectin pathways, thus interfering with the stimulation of the complement system, critical for suppressing viral infection during the initial phase of the innate immune response [21]. Interferons (IFNs) are key players in controlling the initial phase of DENV viral replication [22]. Plasmablasts, activated during primary and secondary infections, play a crucial role in severe dengue and the cross-reactivity of DENV immune responses with other flaviviruses, potentially impacting cross-protection and disease severity [23].

Chaturvedi et al. demonstrated in their study that the blood-brain barrier (BBB) is compromised during DENV infection in experimental animal models, indicating viral invasion [24]. Although initially considered non-neurotropic, recent reports of neurological cases associated with dengue and the detection of the virus in the cerebrospinal fluid (CSF) over the past two decades suggest otherwise [25]. In DENV, the central nervous system (CNS) affected is identified by tests as anti-DENV immunoglobulin (Ig) M or NS-1 in CSF, isolating the virus from CNS and ruling out other causal agents of viral brain illnesses [26, 27].

**Figure 1:**
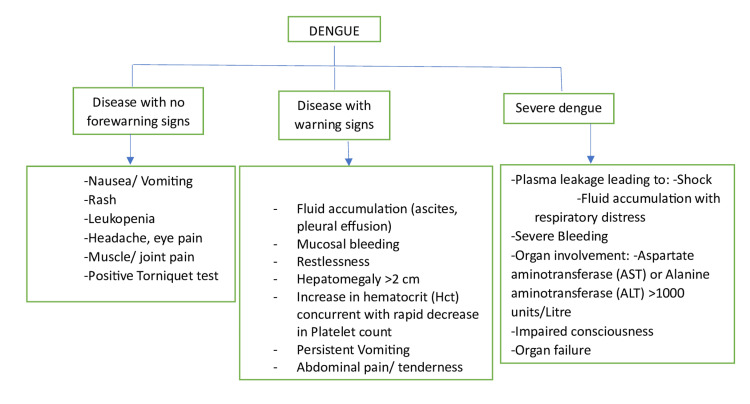
A summarized flowchart of the dengue infection classification Image credit: author Jay P. Patel

A study by Misra et al. on 116 patients, including 82 with dengue fever, revealed CNS involvement in 92 patients, common in DHF/DSS (44%) compared to dengue fever (26%), with worse recovery observed in dengue infection with CNS complications [28]. The most common CNS complications of dengue are encephalitis and encephalopathy [26]. CNS involvement is characterized by impaired mental awareness (Blantyre coma scale <4 for children under five years, Glasgow coma scale <14 for those over five years), neck stiffness, focal neurological signs, or seizures [10]. The World Health Organization (WHO) classifies dengue infection into three categories, summarized in a flowchart (Figure 1).

**Figure 2:**
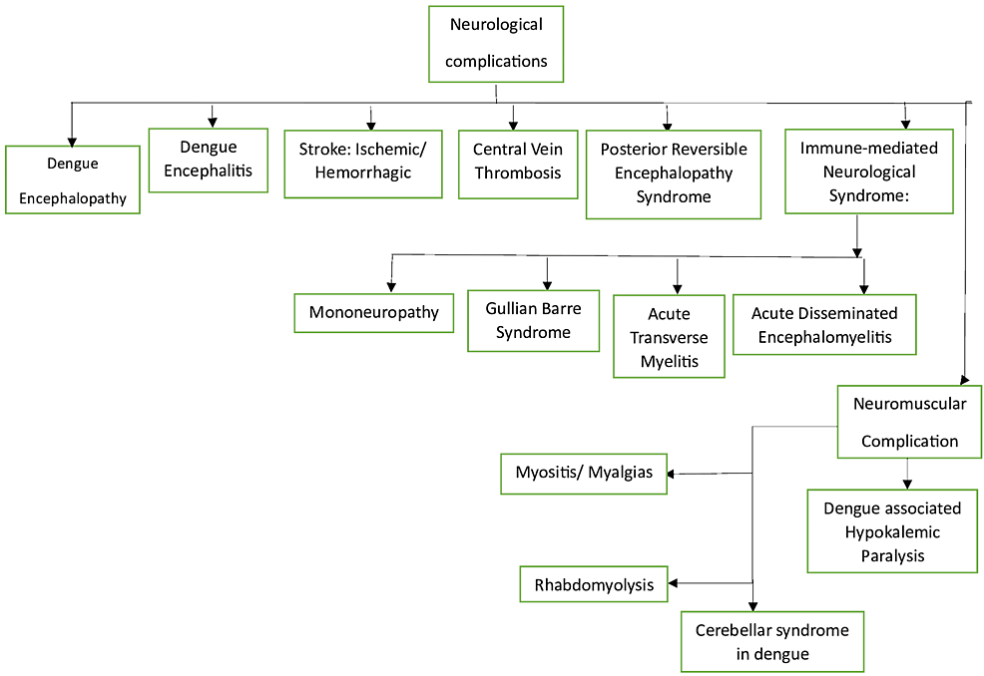
A summarized flowchart of neurological complications in dengue Image credit: author Jay P. Patel

Multiple organs are affected due to severe dengue, including the cardiac, renal, gastrointestinal, respiratory, hematological, and neurological systems. This article delves into the intricacies of neurological complications and challenges resulting from dengue fever. The current classification of neurological complications associated with dengue infection aims to differentiate between the involvement of the central nervous system (CNS) and eyes, peripheral nervous system (PNS) involvement, and immune-mediated illnesses occurring during convalescence or post-dengue recovery [29, 30]. Dengue fever, DHF, and DSS all have the potential to impact the central nervous system [10]. A definitive diagnosis of dengue, as defined by the WHO, is necessary to diagnose any neurological condition caused by DENV [29]. Diagnosis includes any of the following: polymerase chain reaction (PCR) positivity, viral culture positivity, immunoglobulin (Ig) M seroconversion in matched serum samples, or a fourfold rise in IgG titer in paired serum samples [29]. The classification of neurological involvement in dengue fever, presented in the form of a flowchart, is provided below (Figure 2).

Neurological complications affecting the central and peripheral nervous systems have been documented in several studies in India and throughout the world, with an incidence of neurological symptoms ranging from 2.63 to 40% [13, 31].

Dengue encephalopathy

According to the World Health Organization (WHO), encephalopathy is the most commonly encountered neurological complication that may arise from systemic dengue infection [10]. Dengue encephalopathy was previously believed to be solely associated with DHF/DSS [31]. In a prospective case-control study conducted over two years in Vietnam, dengue encephalopathy was encountered in 0.5 percent of 5400 serologically confirmed patients admitted with DHF [32]. Another study prospectively recorded the medical charts of 1493 dengue infection patients in the Department of Pediatrics, Chulalongkorn Hospital, Bangkok, Thailand, from 1987 to 1998. Among them, 46 were diagnosed with dengue encephalopathy, presenting with alterations in consciousness (83.3%), seizures (45.2%), mental confusion (23.8%), nuchal rigidity (21.4%), spasticity of limbs (9.5%), positive clonus (4.8%), hemiplegia (2.4%), and positive Kernig sign (2.4%) [31].

Saluja et al. conducted a study at a government medical college and hospital in Kota and another multi-specialty hospital in Kota from August to November 2017, during a prevalent dengue infection period in the Hadoti region. Among 60 patients with dengue infection presenting neurological symptoms, 30 had encephalopathy, while the remaining patients did not exhibit signs and symptoms of encephalopathy. Of the encephalopathy patients, 16 (53%) experienced seizure, 14 (46%) had respiratory distress, 17 (56%) exhibited shock, three (10%) displayed hemiplegia, and two experienced visual blurring and dysarthria. The mean duration between fever and altered sensorium was recorded as 4.6 (±2.1) days. Among the 30 encephalopathy patients, nine died, while 21 fully recovered, except for three hemiplegic patients. The study concluded that the frequency of dengue encephalopathy has increased in recent years [33].

Dengue encephalopathy typically results in a standard cerebrospinal fluid (CSF) profile; compared to encephalitis, it shows CSF changes due to the direct viral invasion in the brain, and neuroimaging investigations may reveal either normal findings or diffuse cerebral edema [34]. Recent research suggests that the excessive production of cytokines during dengue virus infection triggers immune-mediated damage to endothelial cells within the central nervous system (CNS). Cytokines such as IL-1β, TNF, IL-6, IL-8, and IL-10, along with enzymes like matrix metallopeptidase (MMP2) and chemotactic proteins such as IP2 and RANTES (also known as CCL5), have the potential to induce endothelial injury and dysfunction, leading to heightened vascular permeability and fluid leakage, contributing to cerebral edema [35]. So, basically, dengue encephalopathy is caused by systemic effects and metabolic disturbances.

Dengue encephalitis

Encephalitis is due to the direct viral invasion of the brain and can manifest with altered sensorium or personality, seizures, and localized neurological signs [36]. Over 50 percent of patients may not present with symptoms such as rashes, muscle pain, and bleeding, making diagnosis best achieved through PCR and immunological tests in serum/CSF [10]. Typically, a head CT scan reveals areas of increased density within brain tissue, suggesting spontaneous microhemorrhages, alongside regions of decreased density in the thalami and basal ganglia. Brain MRI is useful in identifying specific anatomical regions of involvement and confirming a diagnosis of dengue encephalitis in individuals exhibiting the aforementioned neurological signs. Commonly affected areas include the basal ganglia, hippocampus, temporal lobes, cerebellum, thalamus, and cerebral white matter, with T2 sequences often showing hyperintensities. Occasionally, similar lesions are detected in the brainstem (especially the substantia nigra) and cerebellum [10, 13].

Meningeal enhancement on post-contrast MRI is infrequent [34]. Diagnosis of DENV-induced CNS illness can be challenging. While identifying dengue NS1 antigen, DENV, and DENV-specific IgM antibodies in CSF can aid in confirming the diagnosis of dengue encephalitis, the test's sensitivity is limited. Similarly, PCR investigations may yield modest results due to decreased CSF virus concentration [10]. Therefore, the diagnosis of dengue encephalitis is typically based on clinical suspicion of dengue, confirmation of systemic DENV infection, manifestation of encephalitic syndrome with or without abnormal cerebrospinal fluid (CSF) findings, and abnormal brain imaging [10].

In 2019, Weerasinghe et al. reported a case involving dengue encephalitis accompanied by status epilepticus. Their findings revealed significant alterations in subcortical white matter and cortical gray matter, with pronounced intensity changes on T2W and FLAIR images [37]. In 2017, Kumar et al. proposed describing the MRI findings of dengue encephalitis as the "double donut sign," observed in a 23-year-old primigravida with a 10-day fever history and three days of impaired sensorium. Lesions in the bilateral thalami appeared hypointense on T1-weighted images and hyperintense on T2W and FLAIR images. On SWI, the lesions showed significant diffusion limitation with hemorrhage [38]. However, most patients typically recover spontaneously with no significant residual deficit.

Central venous thrombosis

Cerebral venous thrombosis (CVT) is a type of cerebrovascular disorder that predominantly affects young individuals, particularly women. Its clinical manifestations can vary widely and may be challenging to identify [12]. Although the clinical presentation is often subacute, approximately one-third of individuals experience an abrupt onset of symptoms. Headache is the most common symptom, affecting more than 90% of patients, and it can be localized or generalized. Around half of CVT patients exhibit focal neurological abnormalities. The signs of parenchymal damage caused by CVT range from cerebral edema to venous infarction and intracerebral hemorrhage [12].

The underlying etiology of CVT remains unknown; however, there is increasing evidence suggesting that inflammation plays a role in the pathophysiology of severe CVT [12]. In a case reported by Vasanthi et al., a 16-year-old boy presented with a fever for two weeks duration, double vision, and headache. On the third day, he developed multiple erythematous rashes all over the body, and an eye examination revealed bilateral papilloedema [39]. Further investigation revealed positive dengue serology and thrombocytopenia (45000/cumm). An MRI brain with a venogram showed bilateral transverse sinus thrombosis [39]. Consequently, he was diagnosed with cerebral venous thrombosis caused by dehydration and an underlying dengue infection. The patient was managed conservatively and hydrated. Two weeks later, a repeat MR venogram showed recanalization of the bilateral transverse sinus [39]. The authors concluded that excessive dehydration leads to cerebral venous sinus thrombosis, emphasizing that adequate hydration in the early phase is crucial to prevent dreaded dengue-related neurological sequelae. Proper hydration may achieve full sinus recanalization without anticoagulation [39]. In another case report, Tilara et al. concluded that while several factors may increase the risk of CVT, dehydration is the primary one [40].

Dengue associated stroke

DENV infection stroke may be ischemic or hemorrhagic [10]. In ischemic stroke, the areas mainly affected are watershed areas, cortical infarction, and lacunar infarction [10]. In hemorrhagic stroke, the hemorrhage could occur in areas of the brain such as the basal ganglia, lobar, cerebellar, pontine, subdural hematoma, subarachnoid hemorrhage, and pituitary apoplexy (hemorrhage) [10].

For confirmed dengue patients who suffer from hemorrhage stroke, the proportion varies from 0.26% (India) to as minimum as 0.06% (Brazil) [10, 41]. The patients appear with fever, moderate to severe headache, vomiting, sudden hemiparesis, and impaired consciousness [42]. The hemorrhage may be caused by elevated vascular permeability, leakage, and vasculitis [42]. They have intracranial bleeding a week after fever onset [43]. Platelet count does not necessarily correspond with the development of intracranial hemorrhage, indicating the potential interaction of numerous variables such as vasculopathy, coagulopathy, and platelet dysfunction [44]. The presence of CSF immune markers in the CSF suggests a disruption of the BBB and blood-CSF barrier in individuals with severe dengue. The NS1 antigen may also stimulate the conversion of plasminogen to plasmin, resulting in fibrinolysis, and for the confirmation of stroke, head CT and brain MRI should be done. There are no evidence-based guidelines for treating dengue-associated cerebral bleeding [10, 44].

Posterior reversible encephalopathy syndrome (PRES)

This condition is seldom encountered in cases of dengue and is identifiable on MRI scans, particularly on T2 and FLAIR sequences. [45]. Bilateral cortical visual loss may be seen in aware individuals or when they regain cognition during recuperation. Its pathophysiology is distinct from those seen in hypertensive crises like eclampsia or pre-eclampsia. The dengue virus infection-associated posterior reversible encephalopathy syndrome (PRES) is cytotoxic rather than vasogenic. Damage to the endothelium is a crucial factor in the emergence of PRES during dengue infection, which explains its reversibility in infection management. Other pathologic processes involved include platelet activation, platelet-activating factor production, and nitric oxide release [45].

Immune-mediated neurological syndromes

Below are descriptions of different immune-mediated neurological syndromes. Typically, post-dengue immune-mediated neurological syndromes resolve within weeks or a few months.

Mononeuropathy

Optic neuritis, oculomotor nerve palsy, isolated sixth nerve palsy, isolated Bell's palsy, long thoracic neuropathy, and isolated phrenic nerve palsy are among the cranial nerve disorders associated with dengue fever. The diagnosis is typically one of exclusion. The predominant pathogenic mechanism is believed to be immune-mediated, and treatment primarily focuses on providing supportive care. Corticosteroids may be beneficial if administered at the outset of the disease [10, 46].

Guillain Barre Syndrome (GBS)

GBS might appear early or late in the illness's progression. The precise pathogenetic process is unknown, although this is highly likely an immune-mediated illness; when dengue-induced immunoglobulins interact with peripheral nerve components containing shared cross-reactive epitopes, it triggers an immune response that may target either the myelin or axons, leading to polyneuropathy typically depicted by demyelination and axonal damage [10].

Acute Disseminated Encephalomyelitis

Acute disseminated encephalomyelitis may occur during the convalescence phase, followed by dengue fever and DHF. The patients present with seizures, altered consciousness, and focal neurological deficits; symptoms usually appear after a febrile period [47]. Perivenous demyelination, infiltration of macrophages, and the presence of lymphocytes surrounding blood vessels with hemorrhagic areas have been documented upon histological examination of these lesions [48]. The pathophysiology was thought to involve a temporary autoimmune response targeting myelin or unidentified self-antigens; for diagnosis, we can use MRI and CSF studies [49].

Acute Transverse Myelitis

Dengue-induced acute transverse myelitis is a relatively rare phenomenon. It may occur during or after the illness. Long-segment engagement is the norm. In the post-infectious phase, the pathogenesis is believed to be immune-mediated, while in the para-infectious phase, it is attributed to direct viral invasion [50]. On spinal cord MR imaging, signal alterations and edema are seen for diagnostic confirmation. Intrathecal production of dengue virus-specific IgG antibodies has been noted in the CSF has been observed, and viral RNA can be isolated [50].

Neuromuscular complications

Dengue-Associated Hypokalemic Paralysis

The start of weakness often comes between the second and fifth days of fever, lasting 4-24 hours. Most patient's muscular stretch responses are missing or diminished. A serum potassium level of 3 mmol/liter or below indicates hypokalemic paralysis [51]. For the pathogenesis of hypokalemia in dengue fever, several mechanisms have been proposed: excess use of intravenous fluids, redistribution of potassium within cells and extracellular fluid, renal tubular abnormalities causing an increase in potassium excretion, catecholamines release due to stress causes cellular uptake of potassium, hypokalemic periodic paralysis has been linked to mutations in the alpha subunit of the L-type calcium channel gene (CACNA1A), and in some instances, mutations in the alpha subunit of the sodium channel gene (SCN4A) [52]. Dengue-associated hypokalemic paralysis is mostly certainly caused by a channelopathy initiated or exacerbated by the virus. Hypokalemic paralysis linked to DENV infection exhibits a swift response to low-dose potassium supplementation, resulting in rapid recovery [52].

Myositis

Dengue-associated myositis might be minor or severe, resulting in quadriparesis and respiratory failure. The proposed mechanisms encompass the invasion of muscle tissues by DENV and immune-mediated damage to muscle fibers, primarily mediated through tumor necrosis factor (TNF) [53, 54]. Histologically, dengue myositis is typified by the infiltration of mononuclear cells around blood vessels, increased mitochondrial activity, accumulation of fat, centralization of nuclei, grouping of muscle fibers based on type, and localized areas of muscle cell death [53, 54]. The treatment of severe myositis using steroids has shown some benefit [10].

Rhabdomyolysis

It is caused by cytokine-mediated damage to the muscle cells. An elevation in cytokine concentrations leads to a rise in intracellular free calcium, which can be attributed to either adenosine triphosphate (ATP) depletion or direct harm and disturbance of the plasma membrane [28]. Elevated levels of intracellular calcium have detrimental effects on muscle cells by inducing the activation of proteases, causing mutations in mitochondria, and leading to an excessive generation of reactive oxygen species. These chemical events ultimately culminate in the breakdown of muscle cells [28]. Further, rhabdomyolysis may cause complications like acute kidney injury and electrolyte disturbances.

Cerebellar Syndrome in Dengue

Cerebellar syndromes, commonly observed in individuals with dengue virus (DENV), are believed to arise from a low-grade inflammatory process that is likely immune-mediated. These syndromes can manifest either in the course of the first stage of the illness or within three weeks following the remission of signs of acute dengue infection and appear to be self-remitting [55].

Myalgia

The early phase of the disease is characterized by muscular soreness, tenderness, and minor muscle edema [56]. The discomfort often impacts the back and proximal limb muscles and results in impaired walking without any accompanying weakening. Direct viral invasion of muscles is probable, leading to subsequent inflammatory alterations and muscular discomfort [56]. The observed histopathological alterations encompass a perivascular mononuclear infiltration of mild-to-moderate intensity, accumulation of lipids, minor proliferation of mitochondria, limited presence of central nuclei, areas of muscle necrosis, and grouping of fiber types. It is transient and self-limiting [56].

Dengue treatment

Common supportive interventions encompass fever management, diligent hematological surveillance, hydration replenishment, and blood or platelet transfusion if necessary [10]. An increase in hematocrit above 20% would indicate substantial fluid loss (plasma), necessitating intensive volume replacement therapy. According to WHO, volume replacement in dengue crystalloids is used [10]. For every 1% of the typical decline in body weight, it is advised to administer 10 ml/kg of replacement fluid in addition to the maintenance fluids prescribed by the conventional weight-based approach. Severe bleeding symptoms may necessitate platelet infusions [10]. As both prolonged shock and fluid overload are linked with high mortality rates, fluid replacement should be monitored closely to ensure that it is done correctly, for the desired time, with the suitable type of fluid.

There is no particular therapy for encephalitis or encephalopathy. Encephalitis treatment focuses on managing viral infection and inflammation, while encephalopathy treatment focuses on correcting systemic metabolic disturbances. Therapies such as intravenous immunoglobulin treatment or high doses of corticosteroids usually have a positive effect on immune-mediated symptoms [10]. Although phase III clinical studies for the TV-003/TV-005 and TAK-003 vaccines are already underway with encouraging findings, Dengvaxia is the only approved DENV vaccine to date [57]. However, as we mentioned, it has been demonstrated that variations in the age and serostatus of the vaccinated individuals directly affect the safety and effectiveness of the vaccine. Dengvaxia and TAK003 (DENVax) are the only DENV vaccines tested in children, with mixed outcomes. Most safety issues surrounding the DENV vaccines stem from phase III pediatric clinical studies [57]. Dengvaxia is based on the yellow fever backbone with an overall efficacy of 30.2-60.8 percent [57]. There is no antiviral drug currently approved for dengue. Vector control is also the mainstay to prevent dengue spread by using mosquito nets, cleaning the dirty water collected at various places, and maintaining a hygienic environment. Strong vector and epidemiological surveillance means that routine control activities can be heightened to specifically target dengue clusters [58]. Community involvement programs to raise awareness are supplemented with house-to-house mosquito habitat inspections and a regulatory framework that includes fines for insubordination [58]. The Singapore dengue control effort teaches valuable insights that may be applied to other Aedes control initiatives and vector control programs more broadly. Since initiating vector control operations in the 1960s, the Singapore dengue control program has reduced the dengue force of infection tenfold by the 1990s and has kept it low ever since [58]. Consideration of dengue as an environmental disease is critical to this achievement, with a heavy emphasis on source reduction and other environmental management measures as the primary vector control strategy [58].

Challenges faced in dengue

Increased disease severity in older age groups and severe comorbidities. Also, there is an increase in infection in pregnant women with higher morbidity and mortality accompanied by adverse fatal outcomes [59]. Increased incidence of dengue and longer dengue seasons. There is cross-reactivity IgG with other flaviviruses in serological assays and a lack of biomarkers that could be used in early illness to predict severe dengue [59]. Furthermore, many adequately conducted studies have not looked into such markers in early disease or during the critical period when leakage starts [60]. The lack of knowledge of the particular mediators that cause vascular leakage has impeded the development of potential therapies as well as the creation of a reliable biomarker that can identify who is likely to develop leakage during the early stages of sickness. Because the clinical manifestations of dengue are so dynamic, it is vital to conduct an unbiased investigation of inflammatory mediators that may induce leakage during the febrile and critical phases [60]. Swaminathan et al. reported a unique case of pregnant women with one day of fever and normal blood pressure and no other symptoms. Two days later, she came back with features suggesting pre-eclampsia with Hemolysis, Elevated Liver enzymes and Low Platelets (HELLP) syndrome and underwent an emergency C-section, delivering a healthy baby at 37+6 weeks of gestation [61]. On day two, the baby developed a seizure, fever, and respiratory distress and was admitted to the neonatal intensive care unit (NICU). Later, various tests found that NS-1 antigen by ELISA and IgM levels were increased in paired titre. Treatment was given according to the symptoms and suspected as congenital dengue in the neonate [61]. As the condition worsened and after giving treatment, weaned off the oxygen. She was then discharged and followed until one month [61].

Strengths and limitations

Our article aims to encompass the neurological complications of dengue because of it's dynamic nature and myriad etiological sources. Our focus remains primarily on facilitating rapid review and study; thus, the scope is limited to neurological manifestations. Notably, central nervous system (CNS) complications associated with dengue have exhibited an upward trend in recent years, indicating the imperative for further investigation into the underlying pathological mechanisms. It is evident that there is currently no specific treatment regimen established for managing dengue-related encephalopathy or encephalitis, thereby highlighting the urgent need for additional research endeavors aimed at mitigating the neurological sequelae of dengue infection.

## Conclusions

Accurately diagnosing acute febrile illnesses in tropical/subtropical regions presents a formidable challenge. However, advancements in immunodiagnostic techniques and the widespread availability of molecular diagnostic tools have somewhat mitigated this challenge. The complexity of diagnosis escalates when neurological manifestations, such as significantly altered sensorium, accompany acute febrile illnesses. Furthermore, distinguishing between dengue encephalopathy and encephalitis poses a considerable challenge. In suspected cases of dengue fever, thorough evaluation of cerebrospinal fluid (CSF) in febrile patients with impaired awareness is crucial to exclude underlying central nervous system (CNS) pathologies. Adequate hydration is imperative to prevent further CNS complications, given the susceptibility of dengue patients to dehydration, while regular monitoring of complete blood count aids in the early detection of changes. Furthermore, extensive research endeavors and a holistic approach are warranted to address the multifaceted concerns of individuals afflicted with dengue fever. Accurate interpretation of neurological manifestations, alongside CSF examination and magnetic resonance imaging (MRI) of the brain and spinal cord, are pivotal for categorizing neurological complications associated with dengue fever.

This literature review serves as a guide to navigating the challenges posed by dengue CNS complications, offering diverse strategies to understand the pathogenesis, diagnosis, and treatment in a timely manner. Providing valuable insights for students and physicians, this article endeavors to enhance understanding of the neurological complications arising from dengue fever and aims to facilitate timely intervention to prevent complications.
